# Mechanical Thrombectomy as Definitive Therapy for Proximal Pulmonary Embolism Post Cardiac Arrest

**DOI:** 10.7759/cureus.102417

**Published:** 2026-01-27

**Authors:** Ahmed Hussein, Badrinathan Chandrasekaran, Paul Foley, Steve Ramcharitar

**Affiliations:** 1 Cardiology, Great Western Hospitals NHS Foundation Trust, Swindon, GBR

**Keywords:** cardiopulmonary arrest, haemodynamic stability, intermediate-high- and high-risk pulmonary embolism, left ventricular ejection fraction (lvef), massive pulmonary embolism, pulmonary artery thrombectomy, right ventricular free wall longitudinal strain, tapse score

## Abstract

Acute pulmonary embolism remains a major cause of cardiovascular morbidity and mortality, with presentations ranging from stable exertional breathlessness to rapidly fatal hemodynamic collapse. In patients who fall in the intermediate high-risk and high-risk categories, prompt restoration of pulmonary perfusion is crucial to reduce right ventricular dysfunction and prevent clinical deterioration. Systemic thrombolysis can achieve rapid reperfusion but carries a substantial risk of major bleeding and intracranial hemorrhage, particularly in older adults or those with comorbidities. This therapeutic limitation has driven increasing interest in mechanical thrombectomy, a catheter-based intervention that offers rapid clot debulking while minimizing hemorrhagic risk. We report a case of high-risk acute pulmonary embolism, which led to hemodynamic compromise and cardiopulmonary collapse, in which systemic thrombolysis failed to achieve adequate reperfusion and correction of acute hypoxia. The patient was successfully treated with catheter-directed aspiration thrombectomy. This highlights the need for consideration of mechanical thrombectomy as a fast and efficient treatment for selected patients who fall into intermediate and high-risk categories, especially as the technology and experience develop.

## Introduction

Acute pulmonary embolism leads to an abrupt increase in pulmonary vascular resistance and right ventricular afterload, and when significant enough, can result in hemodynamic instability. High-risk pulmonary embolism is a dire cardiovascular emergency and portends a poor prognosis. Traditional therapeutic options for rapidly reducing thrombus burden, such as systemic thrombolysis and surgical pulmonary endarterectomy, have limitations regarding both appropriate candidates and efficacy, and there is limited data demonstrating their benefit in high-risk pulmonary embolism. There are advancing percutaneous treatment options that include both localized thrombolysis and mechanical embolectomy.

Despite the growing adoption of mechanical thrombectomy, optimal patient selection continues to be under study with mixed clinical outcomes. In high-risk patients, it is an effective lifesaving strategy in selected patients who can tolerate the procedure [1] and in whom the thrombus location is in an ideal retrieval position for effective thrombectomy and minimal procedural complications [2].

We present a case of acute pulmonary embolism that led to out-of-hospital cardiac arrest. Mechanical thrombectomy was performed after failure of thrombolysis to stabilize the patient, resulting in immediate hemodynamic improvement and eventually a successful discharge.

## Case presentation

A 75-year-old African female with a background of hypertension and poorly controlled diabetes presented with an out-of-hospital cardiac arrest with return of spontaneous circulation after cardiopulmonary resuscitation (CPR) after one minute of chest compressions.

**Figure 1:**
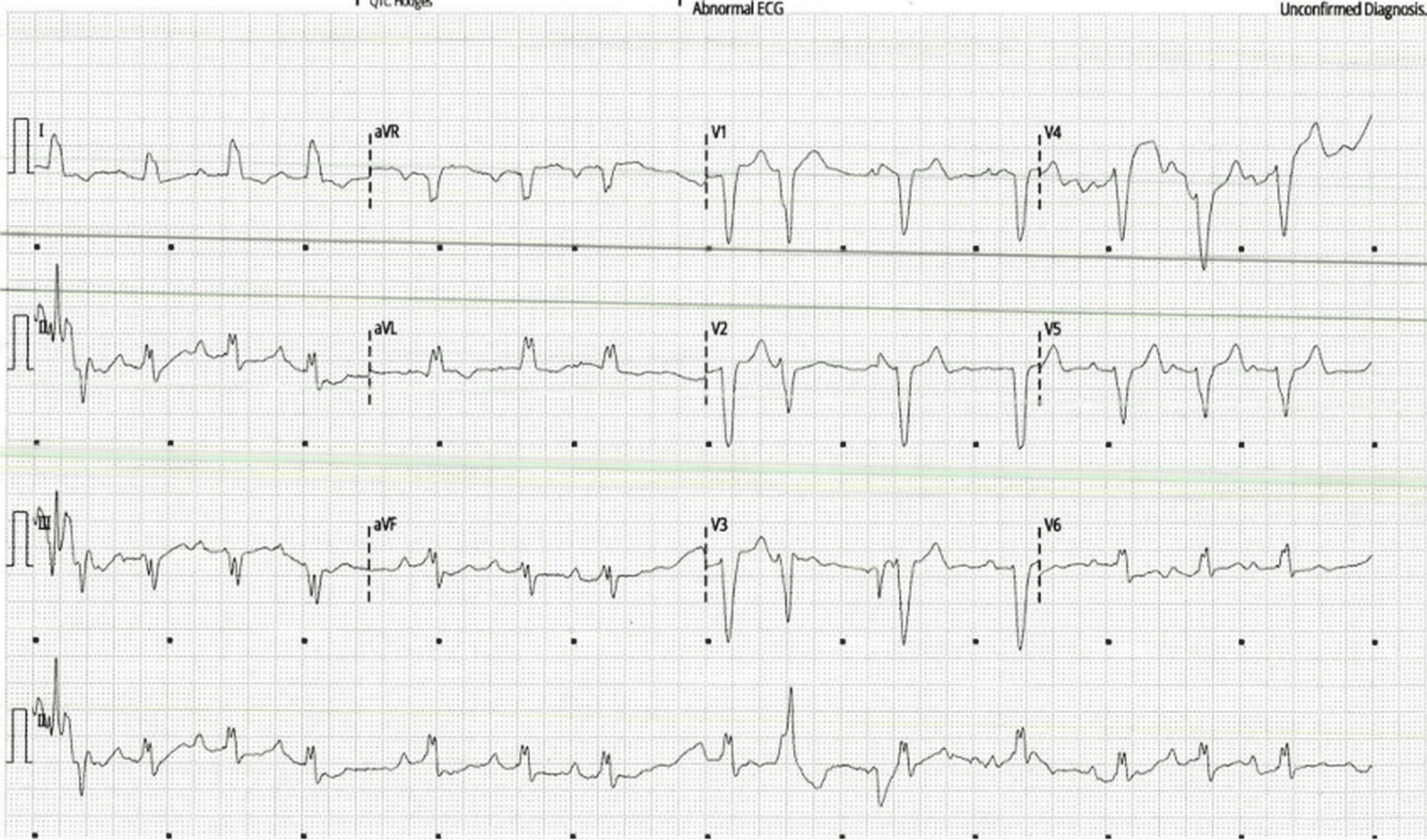
Admission electrocardiogram showing sinus arrhythmia with left bundle branch block

On initial assessment, she was conscious; however, she was hemodynamically unstable with blood pressure (110/77 mmHg), tachycardia (120 beats per minute), hypoxia with peripheral oxygen saturation (SpO₂) of 76% on room air, and confusion indicative of pre-shock. Physical examination revealed features of right-sided heart failure, raised jugular venous pressure, and a loud second heart sound with increased use of accessory muscles of respiration. She gave a four-day history of exertional dyspnea and chest pain. The electrocardiogram showed sinus arrhythmia with a left bundle branch block pattern (Figure 1). Admission laboratory investigations demonstrated significantly elevated cardiac biomarkers, with high-sensitivity troponin of 113 ng/L (normal <14 ng/L) and B-type natriuretic peptide (BNP) of 1589 pg/mL (normal <100 pg/mL). Renal function was impaired, with a creatinine of 207 µmol/L (normal 49-90 µmol/L) and an estimated glomerular filtration rate of 20 mL/min/1.73 m² (normal ≥90 mL/min/1.73 m²). Serum lactate was elevated at 3.6 mmol/L (normal 0.5-2.0 mmol/L), consistent with systemic hypoperfusion. Hemoglobin was 118 g/L (normal 115-165 g/L for females).

**Figure 2:**
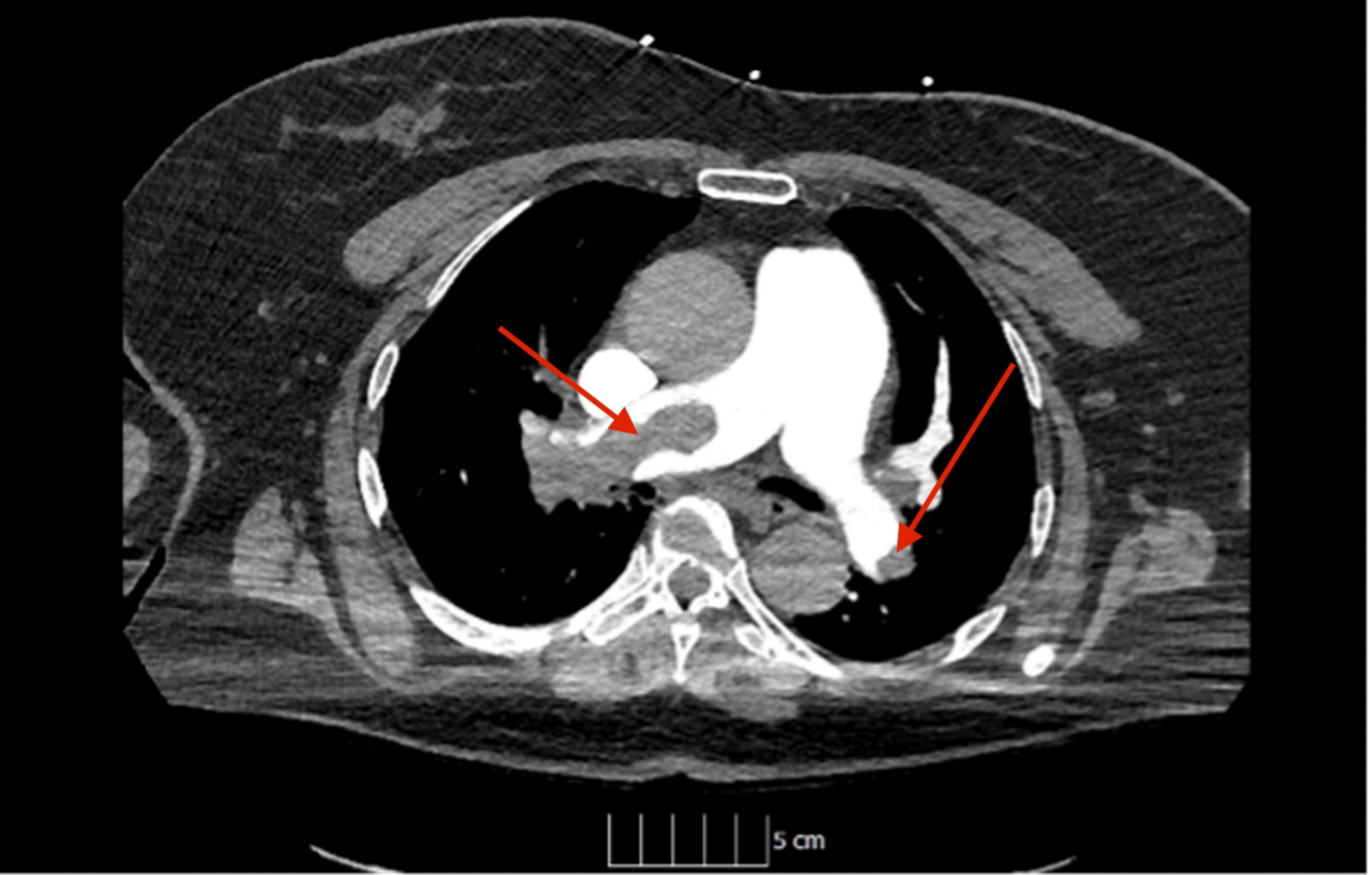
Axial images of a computed tomography pulmonary angiogram Showing bilateral pulmonary embolism with occlusive thrombus in the right main pulmonary artery (left arrow) and likely non-occlusive in the left main pulmonary artery (right arrow).

A subsequent CT pulmonary angiogram revealed a large volume bilateral proximal pulmonary embolism with right heart strain and a right ventricular-to-left ventricular (RV/LV) diameter ratio of >1 (Figure 2).

Given the diagnosis of acute pulmonary embolism and hemodynamic instability, she was given half-dose thrombolysis (Alteplase 50 mg as per our trust's guidelines). The decision for half-dose thrombolysis was made to minimize the risk of major bleeding and intracranial hemorrhage [3]. She was also commenced on antibiotics for a lower respiratory tract infection.

After 48 hours, despite thrombolysis, the patient remained hypoxic, requiring 3 liters of oxygen, and was still in a decompensated cardiac state. Echocardiogram revealed severe bi-ventricular dysfunction (left ventricular systolic dysfunction with an ejection fraction of 32%, reduced tricuspid annular plane systolic excursion of 1.1 cm (normal > 1.6 cm), and severely impaired right ventricular free-wall longitudinal strain of −7.7% (normal < −18%)).

According to the European Society of Cardiology pulmonary embolism guidelines, the patient fell into the high-risk category, and mechanical thrombectomy was indicated (Class IIA).

**Figure 3:**
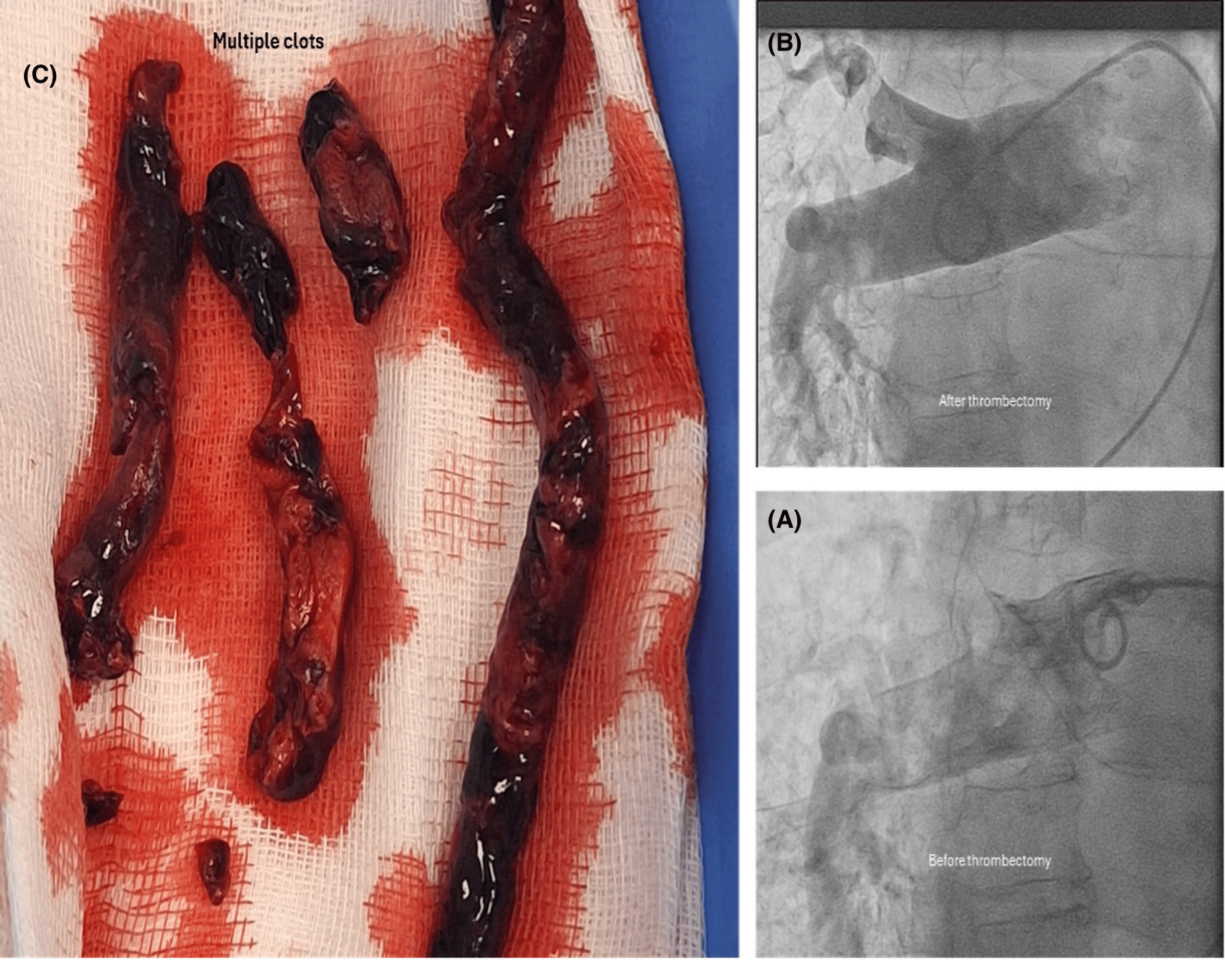
Right heart catheterization fluoroscopy images Revealing improvement of pulmonary artery perfusion post-mechanical thrombectomy (A and B) and the large thrombus retrieved during the procedure (C).

**Figure 4:**
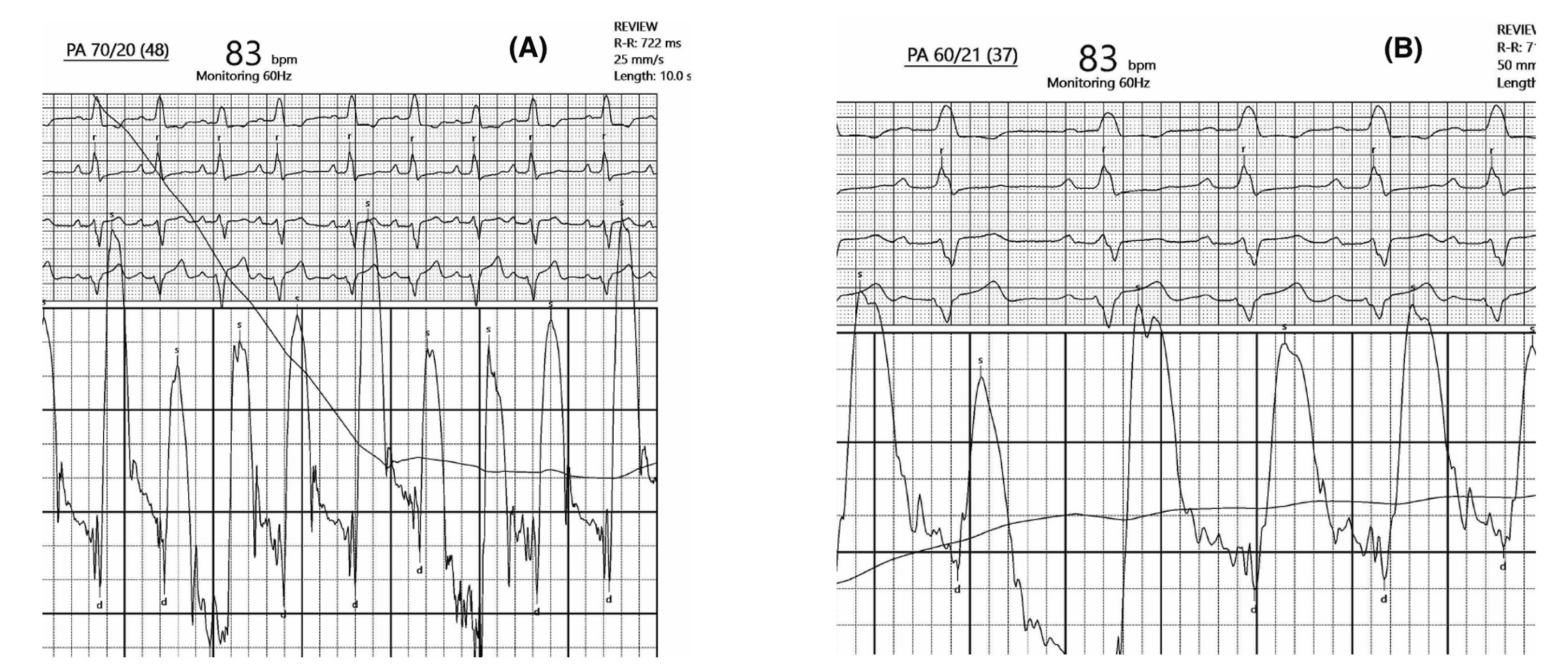
Right heart catheterization Showing improvement of pulmonary artery pressures from 70/20 mmHg (A) to 60/21 mmHg (B) post-thrombus retrieval.

During mechanical thrombectomy, the initial pulmonary artery (PA) pressure was 70/20 mmHg (mean 48 mmHg), and a significant clot burden was present. The PA pressure improved significantly to 60/21 mmHg (mean 37 mmHg) following thrombus retrieval, with improved pulmonary perfusion on fluoroscopy images. The procedure was performed using the FlowTriever mechanical thrombectomy system (Inari Medical, CA, USA) through right femoral venous access with a 6-French sheath. The total procedure time was approximately two hours. There were no immediate complications or major adverse events within 48 hours of the procedure (Figures 3, 4).

**Figure 5:**
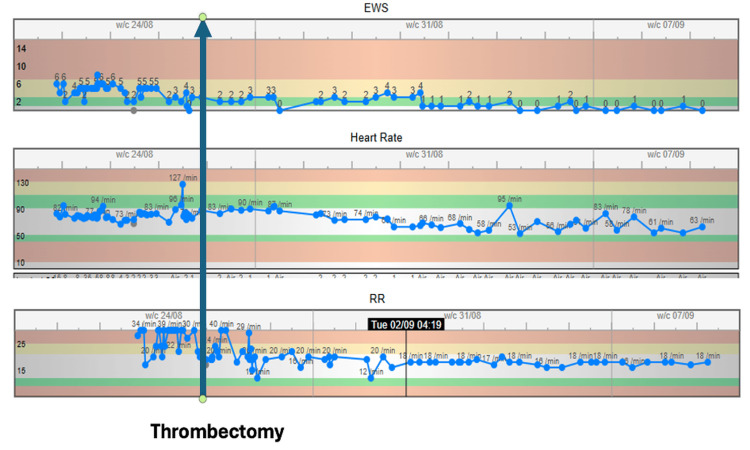
National Early Warning Score chart Showing improvement of tachycardia and tachypnea post-mechanical thrombectomy [4].

**Figure 6:**
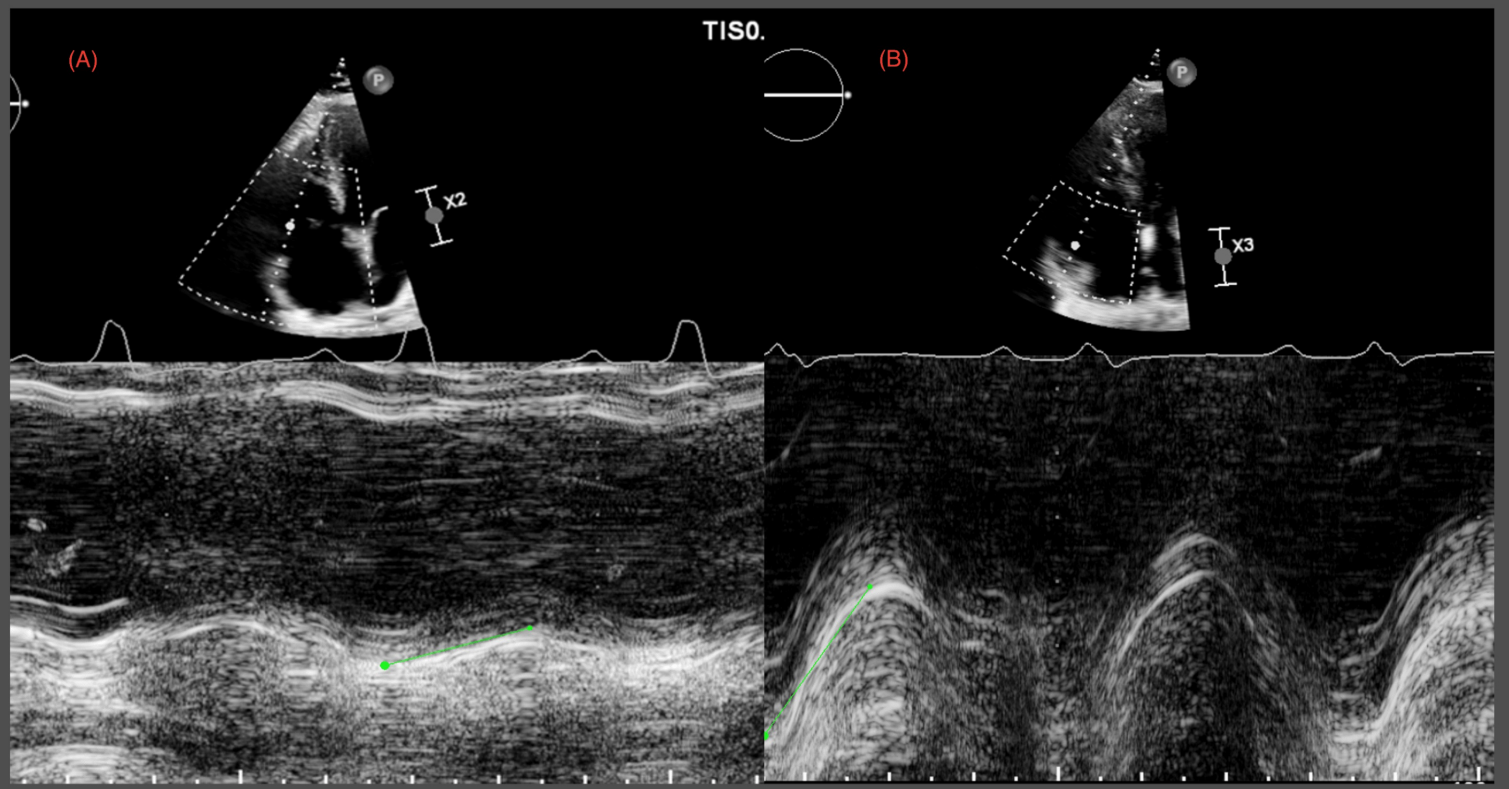
Echocardiography images Before thrombectomy (A) and after thrombectomy (B), revealing normalization of tricuspid annular plane systolic excursion from 1.10 cm to 2.3 cm.

Within 24 hours of the procedure, the patient’s symptoms significantly improved. She was only complaining of mild dyspnea on exertion, but was able to remobilize independently. Oxygen was successfully weaned off over 48 hours, and resolution of the tachycardia was noted (Figure 5). An echocardiogram was repeated 48 hours post-procedure, which revealed normalizing of right ventricular function (tricuspid annular plane systolic excursion improved to 2.3 cm (normal > 1.6 cm), with right ventricular free-wall longitudinal strain of −16.5% (normal < −18%)) and significant left ventricular impairment (ejection fraction = 32%) (Figure 6). The reason for left ventricular failure is unclear; however, it is likely pre-existing cardiomyopathy. The fact that the right ventricular function has normalized post-procedure makes myocardial stunning less likely. Accordingly, she was commenced on optimized heart failure medication and was deemed medically optimized for discharge on day 7 of admission. Following up with the patient, she was still alive at 30 days post discharge and stated that she is back to her baseline functional capacity. 

## Discussion

Massive pulmonary embolism represents less than 10% of all acute pulmonary embolism cases and is a medical emergency with a high mortality rate. The primary treatment for pulmonary embolism is systemic anticoagulation. In cases of massive or submassive pulmonary embolism, systemic thrombolysis may be administered, and in rare instances, open surgical embolectomy is performed [1]. Catheter-directed therapies, such as catheter-directed thrombolysis (CDT) and percutaneous thrombectomy, may also be utilized. Percutaneous thrombectomy is typically used for patients with massive pulmonary embolism who cannot receive thrombolysis due to contraindications, when thrombolysis has failed, or when surgery is contraindicated.

In the latest European Society of Cardiology guidelines (2019), mechanical thrombectomy has emerged (Class IIA) as a promising strategy for selected patients presenting with hemodynamically unstable proximal acute pulmonary embolism who are not suitable for thrombolysis, exhibit right ventricular dysfunction, and have a high pulmonary embolism severity index (PESI) score during the acute phase.

Early prospective trials such as FLARE (FlowTriever Pulmonary Embolectomy Clinical Study) and EXTRACT-PE (Evaluating the Safety and Efficacy of the Indigo Aspiration System in Acute Pulmonary Embolism) demonstrated a significant reduction in RV/LV ratio within 48 hours, with very low rates of major bleeding [5-7]. This supports the feasibility and safety of mechanical thrombectomy in intermediate- and high-risk pulmonary embolism. Large real-world registries, including FLASH (FlowTriever All-Comer Registry for Patient Safety and Hemodynamic Efficacy) and FLAME (FlowTriever for Acute Massive Pulmonary Embolism), further confirmed hemodynamic benefit and low early mortality in these cohorts [7,8]. The PEERLESS trial (Pulmonary Embolism Revascularisation Using Large-Bore Mechanical Thrombectomy Versus Catheter-Directed Thrombolysis) demonstrated that, in patients with high-risk pulmonary embolism undergoing catheter-based intervention, large-bore mechanical thrombectomy resulted in less frequent clinical deterioration and lower post-procedure intensive care unit (ICU) utilization, with a comparable bleeding risk to CDT [8-10]. In addition, several studies have demonstrated a notable reduction in hospital length of stay following mechanical thrombectomy, which not only decreases the risk of hospital-acquired infection and overall healthcare costs but also has a positive impact on patients’ psychological well-being [11].

In our case, where thrombolysis alone had failed, mechanical thrombectomy was successful, resulting in a significant reduction of PA pressures, hemodynamic stabilization, and immediate improvement in symptoms. Furthermore, we were able to facilitate an early discharge following optimization of her heart failure medication.

In conclusion, large-bore mechanical thrombectomy represents a vital, life-saving intervention for patients with high-risk pulmonary embolism, particularly those who have failed systemic thrombolysis. When performed in clinically suitable candidates, this approach provides rapid hemodynamic stabilization and offers a definitive therapeutic pathway in the acute setting.

## Conclusions

This case highlights the pivotal role of mechanical thrombectomy as an effective rescue strategy in selected patients with high-risk pulmonary embolism who demonstrate persistent hemodynamic compromise despite systemic thrombolysis. The intervention resulted in a rapid reduction of pulmonary artery pressures, marked improvement in right ventricular function, and sustained clinical stabilization without procedural complications. These findings reinforce the growing evidence supporting mechanical thrombectomy as a valuable component of the contemporary management algorithm for acute pulmonary embolism, particularly in patients with contraindications to, or failure of, thrombolytic therapy. Careful patient selection within a multidisciplinary framework remains essential to optimize outcomes and maximize procedural benefit.
